# Topography of connections between human prefrontal cortex and mediodorsal thalamus studied with diffusion tractography

**DOI:** 10.1016/j.neuroimage.2010.02.062

**Published:** 2010-06

**Authors:** Johannes C. Klein, Matthew F.S. Rushworth, Timothy E.J. Behrens, Clare E. Mackay, Alex J. de Crespigny, Helen D'Arceuil, Heidi Johansen-Berg

**Affiliations:** a Centre for Functional MRI of the Brain, University of Oxford, Oxford, UK; b Department of Neurology, Goethe-University of Frankfurt, Frankfurt/Main, Germany; c Department of Experimental Psychology, University of Oxford, Oxford, UK; d Department of Psychiatry, University of Oxford, Oxford, UK; e Athinoula A. Martinos Center for Biomedical Imaging, Department of Radiology, Massachusetts General Hospital, Charlestown, MA 02129, USA

**Keywords:** Anatomy, DTI, Human, Macaque, Thalamus

## Abstract

Studies in monkeys show clear anatomical and functional distinctions among networks connecting with subregions within the prefrontal cortex. Three such networks are centered on lateral orbitofrontal cortex, medial frontal and cingulate cortex, and lateral prefrontal cortex and all have been identified with distinct cognitive roles. Although these areas differ in a number of their cortical connections, some of the first anatomical evidence for these networks came from tracer studies demonstrating their distinct patterns of connectivity with the mediodorsal (MD) nucleus of the thalamus. Here, we present evidence for a similar topography of MD thalamus prefrontal connections, using non-invasive imaging and diffusion tractography (DWI–DT) in human and macaque. DWI–DT suggested that there was a high probability of interconnection between medial MD and lateral orbitofrontal cortex, between caudodorsal MD and medial frontal/cingulate cortex, and between lateral MD and lateral prefrontal cortex, in both species. Within the lateral prefrontal cortex a dorsolateral region (the principal sulcus in the macaque and middle frontal gyrus in the human) was found to have a high probability of interconnection with the MD region between the regions with a high probability of interconnection with other parts of the lateral prefrontal cortex and with the lateral orbitofrontal cortex. In addition to suggesting that the thalamic connectivity in the macaque is a good guide to human prefrontal cortex, and therefore that there are likely to be similarities in the cognitive roles played by the prefrontal areas in both species, the present results are also the first to provide insight into the topography of projections of an individual thalamic nucleus in the human brain.

## Introduction

There is clear evidence that a fundamental distinction can be drawn between two principal networks of medial and orbitofrontal cortical areas in both rats (Floyd et al., 2000, 2001; Ray and Price, 1992, 1993) and macaques (An et al., 1998; Carmichael and Price, 1995a,b, 1996; Hsu et al., 2008; Kondo et al., 2005; Ongur et al., 1998). There is evidence for a third group of frontal areas in lateral prefrontal cortex that are most prominent in primates (Murray et al., 2000; Passingham et al., 2000).

The first network is centered on central and lateral orbitofrontal cortex and, in the macaque, is interconnected with cortical regions processing sensory information in many modalities. The second network is centered on medial frontal and cingulate areas and is interconnected with visceromotor and motor structures. The orbital cortex of both rats and macaques is critical for learning about reward-predicting sensory stimuli and for representing reinforcement expectations (Murray et al., 2007; Rushworth et al., 2007a,b). By contrast, medial frontal areas are more concerned with reward-guided action selection, the representation of action values, and the valuation of social information in both rats and macaques (Rudebeck et al., 2008, 2006, 2007).

On the basis of comparative cytoarchitectonic studies and investigations of sulcal patterning it has been argued that similar orbital and medial areas can be identified in the human brain (Chiavaras et al., 2001; Chiavaras and Petrides, 2000; Öngür et al., 2003; Petrides and Pandya, 2002). The functions of specific cortical areas are constrained and determined by their anatomical connections but little is known of the connections of the human medial and orbital networks and so the degree to which human medial and orbital networks should be expected to have functions resembling those of other mammals is unclear.

Although medial and orbitofrontal networks differ in a number of their cortical connections, differences in connection patterns with the mediodorsal (MD) thalamus provided some of the first evidence for separate medial and orbital networks (Ray and Price, 1992, 1993). Moreover, connectivity between MD thalamus and prefrontal cortex has had an important historical influence on thinking about prefrontal function (Preuss, 1995). Therefore in the present investigation we used diffusion-weighted imaging (DWI) and probabilistic diffusion tractography (DT) to examine whether evidence for a similar pattern of MD–prefrontal connectivity could be found in the human brain.

The third group of frontal areas, forming the lateral prefrontal cortex, is essential for the representation of state contingent action selection rules (Murray et al., 2000; Passingham et al., 2000) and the monitoring of state transitions in order to ensure that the best action is selected in any given environment (Lee et al., 2007; Petrides, 2005). Lateral prefrontal areas are interconnected with yet another distinct MD region (Ray and Price, 1993). There are variations in the extent, and even the presence, of the lateral prefrontal area in various mammalian species (Preuss, 1995). We also used DWI–DT to look for evidence for an MD zone with a high probability of connection with human lateral prefrontal cortex.

**Fig. 1:**
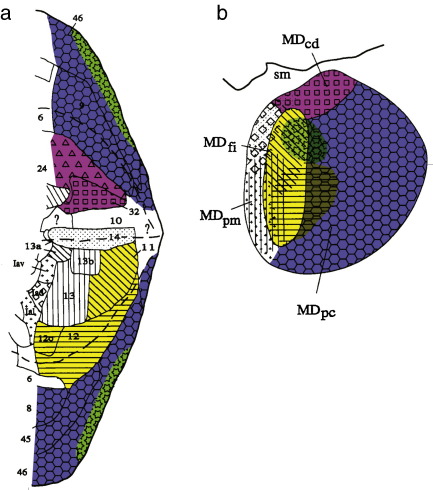
Tracer study of connections between MD and PFC in macaque. (a) Macaque prefrontal cortex is illustrated as a flattened representation, with the green edges intended to join up in a cone-like fashion and form the bottom of the principal sulcus. Based on Ray and Price (1993) with color coding added as follows: DPFC/VLPFC in blue, DLPFC in green, LOFC in yellow, and ACC in magenta. (b) Coronal slice through rostral MD in macaque showing connectivity targets of prefrontal cortex as established by Ray and Price, using the color code introduced in a).

Previous DWI–DT studies have identified regional variations in corticothalamic connectivity (Behrens et al., 2003a; Johansen-Berg et al., 2005) but the present study is the first DWI–DT study to attempt to identify sub-nuclear differences in thalamocortical interconnectivity. DWI–DT suffers from many limitations in comparison to tracer labelling studies that can only be performed in animal models. DWI–DT reveals neither the fine details of synaptic connections nor the direction of projections. Nevertheless it is possible to compare our estimates of regional differences in interconnectivity with those derived from macaque tracer labelling studies (Fig. 1).

## Methods

### Macaque data acquisition

Animal procedures were conducted with approval of the local committee on research animal care. A post-mortem macaque (*Macaca fascicularis*) brain was obtained from a previous study. The animal in question had undergone a 1 hour intravascular middle cerebral artery occlusion experiment and had sustained no permanent lesion as detectable using *in vivo* DWI. Following this the brain was removed after euthanasia and immersed in a 10% formaldehyde solution at 4 °C for at least 4 weeks. Following fixation, it was placed in a solution of 2% phosphate-buffered saline (PBS) doped with 1 mMol/l Gd-DTPA (Magnevist, Berlex Imaging, Montville, NJ, USA) contrast agent. The brain was then mounted on an individually modelled mould and immersed in Fomblin LC/8 (Solvay Solexis Inc., Thorofare, NJ, USA) for susceptibility matching.

High angular and high spatial resolution diffusion-weighted images were acquired using previously described methods (D'Arceuil et al., 2007). Imaging was performed on a 4.7 T/33 cm bore Oxford magnet equipped with BGA 12 gradients interfaced to a Bruker Biospec Avance console (ParaVision 3.0.1; Bruker Biospin, Ettlingen, Germany), using a standard 72 mm inner diameter birdcage coil (Bruker). DWI was performed using a 3D segmented spin-echo EPI sequence (430 µm isotropic resolution, TE 33 ms, 8 shots, TE 33 ms, TR 350 ms, 120 isotropically distributed diffusion directions, *b*_max_ = 8000, delta 6.85 ms, Delta 10.8 ms, 27 h total scan time).

### Human data acquisition

Diffusion imaging in healthy volunteers was approved by the local research ethics committee. Eight healthy volunteers (age range 21–34, four men, four women) underwent DWI scanning on a 1.5 T Sonata MR scanner (Siemens, Erlangen, Germany) using the standard quadrature head coil supplied with the system. Subjects were resting with their head fixed using wedge-shaped foam padding. Diffusion was measured in 60 isotropically distributed directions using echo-planar imaging (SE-EPI, TE 97 ms, TR 10.1 s, 72 axial slices, voxel size 2 mm ∙ 2 mm ∙ 2 mm) using a *b*-value of 1000 s mm^− 2^. To increase signal to noise ratio (SNR), scanning was repeated three times for off-line averaging, requiring a total scanning time of approximately 45 min. Additionally, T1-weighted images (3D FLASH, TE 5.65 ms, TR 12 ms, FA 19°, 256 axial slices, voxel size 1 mm ∙ 1 mm ∙ 1 mm) were recorded to facilitate spatial normalization and for visual assessment of individual anatomy. A further set of proton-density MR images (TR = 6 s, TE = 9.5 ms, in-plane resolution 0.8 × 0.8 mm, slice thickness = 2 mm, coronal slices), obtained from a separate group of 6 healthy adults (3 female, 3 male) on the same scanner, were used to define a mediodorsal nucleus mask. These images were optimised for visualisation of intrinsic thalamic structure (Devlin et al., 2006).

### Data processing

#### Common space

Diffusion tractography (methods detailed below) was carried out in the untransformed space native to macaque or human subjects. Macaque results were then stored in the same native space. In order to compare results across individual human subjects, the human results were stored in MNI152 standard space for further processing. To transfer individual images into standard space, individual human structural MRIs were skull-stripped using the FMRIB software library (FSL) Brain Extraction Tool (BET) (Smith, 2002) and registered to the skull-stripped registration target in MNI152 space using a multi-scale approach maximising correlation ratio (Roche et al., 1998) between image histograms (Jenkinson et al., 2002; Jenkinson and Smith, 2001) using the FSL linear image registration tool (FLIRT). The MNI152 registration target was defined at the Montréal Neurological Institute, averaging 152 stereotaxically normalized T1-weighted images of normal human brains. A copy is provided with the FSL software library (Brett et al., 2001; Smith et al., 2004) (http://www.fmrib.ox.ac.uk/fsl).

For diffusion data, FLIRT was employed to establish registration parameters between a non diffusion-weighted reference volume and each individual anatomical MRI. The registration parameters that were derived were then concatenated with those transforming each individual structural MRI scan into standard space, resulting in transformation parameters taking individual DWIs into standard space.

#### Common space target mask generation

We define a number of ROIs (described in detail below). ROIs for the macaque brain were defined on the individual image with no diffusion weighting. Human ROIs were drawn in each individual scan's native space, registered into standard space (using the registration principles outlined above), then summed across subjects. A threshold was applied to only keep voxels identified as belonging to the region in question in at least 30% of subjects studied. The masks were then binarised, resolving ambiguities at voxels on the borders between regions by assigning the label of the mask in accordance with whether the voxel was included in the mask of the majority of subjects to create binary, mutually exclusive ROIs.

#### Definition of prefrontal target areas — macaque

**Fig. 2:**
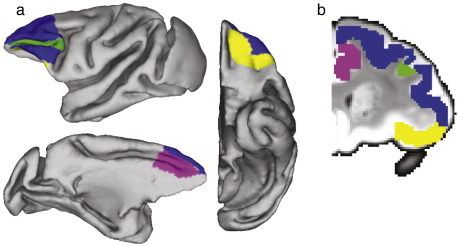
Macaque regions of interest. (a) A 3D illustration of cortical target areas. (b) Coronal slice through cortical target masks. Color coding as for Fig. 1.

Prefrontal cortical target regions were defined on 3D macaque MR images (Fig. 2), following the description of distinctive MD projection zones reported in Ray and Price's (1993) original work (Fig. 1). The caudal border of the entire PFC was defined on a coronal plane rostral to the arcuate sulcus. The rostral border of PFC was on an approximate coronal plane interconnecting the rostral limits of the medial orbital sulcus and the principal sulcus. Specific sub-regions within PFC were then defined on the macaque brain, following the work of Ray and Price (1993) as detailed below.

##### Target for parvicellular part of MD (MDpc): ventral and dorsal parts of the lateral frontal cortex (DPFC/VLPFC)

The parvicellular part of MD (MDpc), which encompasses roughly two thirds of MD's volume on the lateral aspect, projects to the more dorsal and the more ventral PFC regions on the lateral surface including areas 6, 9, 45 and 8 in macaque (Fig. 1, blue). Area 46 in the principal sulcus, however, is not included within the interconnected region even though it lies at the center of the lateral surface between the dorsal and ventral areas. Instead Ray and Price identified a distinctive MD connection pattern for the central dorsolateral region area 46. We therefore refer to the putative target for MDpc as the dorsal PFC/ventrolateral PFC (DPFC/VLPFC) region and distinguish it from the intervening dorsolateral prefrontal PFC (DLPFC). We defined VLPFC as the region extending from the lateral margin of the orbital surface to the ventral lip of the principal sulcus; we defined DPFC as the region extending from the dorsal lip of the principal sulcus to the dorsal lip of the cingulate sulcus (Fig. 2, blue).

##### Target for fibrous part of MD (MDfi): lateral orbitofrontal cortex (LOFC)

Ray and Price (1993) found that macaque lateral orbitofrontal cortex (areas 11 and 12) receives projections from the fibrous part of MD (MDfi), and to a small extent from the medial portion of MDpc (Fig. 1, yellow). Area 11 in macaque, as defined by Brodmann (1905), is located in a relatively rostral position between the lateral and medial orbital sulci in the central orbital region between areas 12 and 14 on the orbital surface. We defined LOFC as the region bounded medially by the medial orbital sulcus and laterally by the most lateral margin of the orbital surface (Fig. 2, yellow). The caudal boundary was shifted rostrally within the region bounded by the medial and lateral orbitofrontal sulci to the transverse orbital sulcus.

##### Target for caudodorsal part of MD: anterior cingulate cortex (ACC)

Ray and Price reported that, in the macaque, ACC connections are especially prominent with the caudodorsal part of MD (MDcd) (Fig. 1, pink). Ray's and Price's investigation was limited to just the most rostral and ventral part of the ACC. When tracer injections were made into MD, Giguere and Goldman-Rakic (1988) demonstrated that more dorsal ACC regions clearly connected to more lateral regions of MD. In the present study we defined the ACC region as including both banks of the cingulate sulcus and the cingulate gyrus and extending as far ventrally as the rostral sulcus and as far caudally as to be level with the bow of the arcuate cortex (Fig. 2, pink). It is therefore to be expected that in the present study evidence for connection with the ACC may extend beyond just a limited region of caudodorsal MD.

##### Area 46 — dorsolateral prefrontal cortex (DLPFC)

Finally, area 46, located within the principal sulcus of the macaque, has no MD thalamus territory that is exclusively devoted to it alone, but instead it receives projections from parts of both MDfi and MDpc (Fig. 1, green). The interconnected region therefore overlaps with regions projecting to DPFC/VLPFC and LOFC. In keeping with Ray and Price's (1993) description of MD projection zones, we defined the DLPFC region as limited to the bottom and banks of the principal sulcus (Fig. 2, green) but not including the more lateral cortex on the surface of the brain.

#### Definition of prefrontal target areas — human

There is some uncertainty about the details of parcellation and labelling of prefrontal regions in the monkey in Brodmann's (1905, 1909) original work. The definitions of frontal cortical target regions in the present study were, therefore, guided by subsequent studies (Pandya and Yeterian, 1996; Petrides et al., 1993; Walker, 1940) that have attempted to highlight similarities in frontal cortex between human and macaque species.

We created probabilistic maps of human prefrontal and anterior cingulate cortex (PFC and ACC) sub-regions based on manually defined individual regions of interest from our previous study (Croxson et al., 2005). In that study, PFC and ACC were subdivided into anatomical regions using landmarks reliably visible on T1-weighted FLASH MRI from 10 healthy volunteers [age range 24–35, six men, four women, (Croxson et al., 2005)]. In brief the PFC/ACC was defined as the region rostral to the superior and inferior precentral sulci on the lateral surface. The caudal limit on the medial surface was defined by a coronal plane 6 mm posterior to the one passing through the anterior commissure on each brain when transformed into standard space. The frontal eye field was excluded from analysis using a standard space mask (Croxson et al., 2005; Paus, 1996), as it is typically considered a premotor rather than prefrontal region. The rostral limit of PFC/ACC was defined as the point where the three lateral sulci could no longer be distinguished clearly. This meant that the most rostral frontopolar cortex was not included in the analysis.

**Fig. 3:**
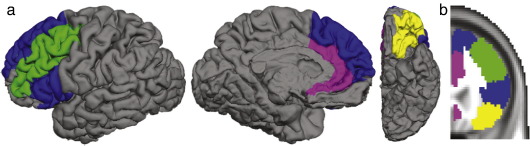
Human regions of interest. (a) A 3D illustration of targets for diffusion tractography, rendered on a single human subject's brain. (b) Coronal slice through the final standard space mask. Color coding as for Fig. 1.

The human PFC/ACC was then divided into four subregions, proposed to correspond to the four cortical regions in macaque identified by Ray and Price (Figs. 1 and 2). We aimed to use unambiguous sulcal landmarks that are reliably visible on MRI scans in order to define the regions that have been hypothesized (Petrides and Pandya, 1994, 1999, 2002), on the basis of cytoarchitectonic considerations, to correspond to the macaque regions of interest. Example cortical masks from a human individual subject are shown in Fig. 3 and mask definitions are described in detail below.

##### Putative target for MDpc: DPFC/VLPFC

DPFC/VLPFC receives projections from MDpc in macaque (Fig. 1). In the human brain, we assigned the superior frontal gyrus, extending from (but excluding) the middle frontal gyrus to the paracingulate or cingulate gyrus to the DPFC region (Fig. 3, blue). The VLPFC region extended from the horizontal ramus of the Sylvian fissure to the inferior frontal sulcus, encompassing the caudal and superior parts of inferior frontal gyrus — the pars triangularis and pars opercularis (Fig. 3, blue).

##### Putative target for MDfi: LOFC

Macaque lateral orbitofrontal cortex (areas 11 and 12) receives projections from MDfi, and to a small extent from the medial portion of MDpc (Fig. 1). The human ventral prefrontal area 47 and the macaque area 12 exhibit topographic and architectonic similarities and it has been suggested that a part of human area 47 corresponds to macaque area 12 and so reference is now often made to area 47/12 in both species (Petrides and Pandya, 1994, 2002). Areas located between the medial and lateral orbital sulci in the human brain have been proposed to correspond to area 11 in macaque (Öngür et al., 2003).

Our human LOFC mask spans the rostral part of the central orbital region and the lateral orbital gyri, located between the horizontal ramus of the Sylvian fissure and medial orbital sulcus (Fig. 3, yellow).

##### Putative target for caudodorsal part of MD: ACC

In macaque, ventral and anterior ACC connections are most prominent in the caudodorsal part of MD (MDcd) (Fig. 1) but Giguere and Goldman-Rakic (1988) have demonstrated that more caudo-dorsal ACC regions are interconnected with a wider part of lateral MD. In the human brains in the present study, the region between the paracingulate sulcus/cingulate sulcus and the corpus callosum was designated anterior cingulate cortex (ACC) (Fig. 3, pink). ACC includes the cingulate gyrus, both banks of the cingulate sulcus, and, when present, the lower bank of the paracingulate sulcus. Rostrally the ACC region extended rostral to the genu of the corpus callosum and then ventrally into the subgenual cortex. The region was therefore likely to include much of areas 24, 25, and 32.

##### Putative DLPFC

Area 46 in macaque receives projections from parts of both MDfi and MDpc (Fig. 1). The human counterpart of area 46 is thought to be found on the middle frontal gyrus (Petrides et al., 1993; Rajkowska and Goldman-Rakic, 1995a,b). In the present study it was defined as the region between the superior and inferior frontal sulcus (Fig. 3, green).

#### Thalamic ROI — macaque

The MD nucleus of the thalamus was visualised and delineated on MR images of diffusion fractional anisotropy. In addition micrographs obtained in various orientations from brainmaps.org were also used as references in order to delineate the MD nucleus.

#### Thalamic ROI — human

We created an ROI of the mediodorsal nucleus based on proton-density weighted images that had been obtained in a separate group of subjects, using a scanning protocol designed for improved intrinsic thalamic contrast (Devlin et al., 2006). MD was outlined in each subject's individual PD-weighted image by reference to the thalamic atlas of Morel, Schaltenbrand and colleagues (Johansen-Berg et al., 2005; Morel et al., 1997; Schaltenbrand et al., 1977). Individual, skull-stripped (Smith, 2002) PD-weighted images were linearly registered to the skull-stripped registration target in MNI152 space using FLIRT. The resulting transformation matrices were applied to individual subject MD nucleus ROIs to align these in standard brain space. Individual MD masks in standard brain space were summed across subjects in order to create a population probability map of MD where voxel values represent the number of subjects in whom the MD is present. This population probability map was thresholded to include only those voxels in which MD was present in at least 50% (i.e., 3/6) of the population.

Supplementary Fig. 1 depicts this mask overlaid onto a slice of Morel's atlas, selected at the mask's center of gravity.

#### DWI analysis

Diffusion data were corrected for eddy currents and, in the case of human subjects, head motion by affine registration to a non diffusion-weighted reference volume (Jenkinson et al., 2002). The three acquisitions collected for each timepoint were averaged to increase SNR. Then, probability distributions of fiber directions were calculated for each brain voxel using a prototype version of a multi-fiber extension (Behrens et al., 2007a) of the probabilistic tractography available in FMRIB's Diffusion Toolbox (Behrens et al., 2003b). In brief, probability distributions of fiber orientation are estimated at every voxel. These distributions' widths correspond to the uncertainty associated with the estimated fiber direction. This uncertainty is not only derived from noise and artifacts in MR imaging, but also from incomplete modelling of the acquired diffusion data, which models two fiber populations in every voxel, even though there may be other co-existing fiber populations. Using this knowledge about local probability distributions, probabilistic diffusion tractography can then estimate the pathways that pass through any given seed voxel, as well as the probability that such a pathway will pass through any other voxel in the brain (Behrens et al., 2003a,b).

Probabilistic multi-fiber diffusion tractography was initiated from every voxel inside the thalamic masks in standard space. With the data acquisition scheme used here, our data processing methods allow for estimation of at most two populations for every voxel, representing the predominant fiber orientations observed within each voxel (Behrens et al., 2007a). 5000 virtual particles were released from each voxel. For voxels in which two fiber directions were estimated, an initial fiber orientation was randomly selected from the two fiber populations estimated at that voxel location. Counters were increased every time a virtual particle struck one of the four target regions, resulting in four images where each thalamic mask voxel's value corresponds to the number of times a virtual particle had reached the target assigned to that volume.

For visualisation, human thalamic connectivity results were summed up for each individual cortical target mask. On the integrated image obtained, representing connectivity likelihood over all subjects studied, the voxel with highest evidence for connectivity, i.e. the highest number of virtual particles striking its cortical target, was located. Then, a threshold was applied removing all those voxels with fewer than 50% of the maximum voxel's connectivity count.

The relative locations of sub-regions within MD connecting to different cortical targets were visually assessed on an individual basis and submitted to statistical testing. Within MD, we recorded the coordinate of the voxel with the highest probability of connectivity to LOFC, DLPFC and DPFC/VLPFC for each subject.

We then performed 3 separate repeated-measures ANOVA on the *X*-component (medial–lateral position), the *Y*-component (rostral–caudal) and the *Z*-component (dorsal–ventral) of these coordinates to test whether the locations of peak MD voxels connecting to different cortical target areas were significantly different from each other along each axis. For any axis for which the ANOVA revealed a significant effect of target, we employed post-hoc paired *t*-tests (one-sided, repeat measures) to test for significant differences in the location of voxels of peak MD connection for each pair of cortical targets. We report statistical parameters (degrees of freedom and *t* value) and the *p* values are calculated.

## Results

### Comparison between macaque tractography results and previous tracer studies

**Fig. 4:**
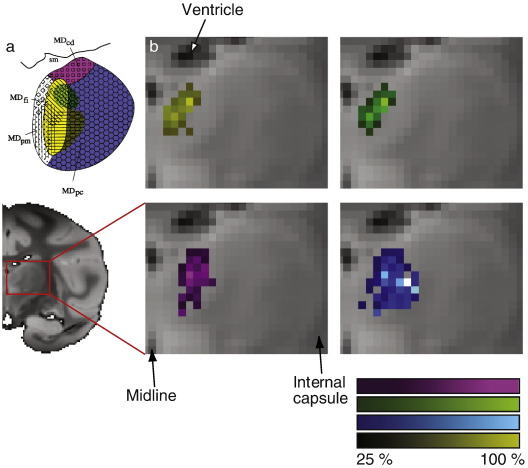
Tractography results in macaque brain. (a) Results from previous tracer study by Ray and Price (1993) repeated from Fig. 1 for reference. (b) Connectivity-based parcellation of macaque MD in a single animal. Voxels within MD are color-coded according to their probability of connection with different cortical areas (shown in Fig. 2). The color bars present color shades used to illustrate connectivity probability as a percentage of the maximum probability of connectivity observed for every target mask.

The topography of tractography-derived prefrontal connections with MD in macaque (Fig. 4) was mostly comparable with the results from tracer injection studies that have previously been conducted in the same species (Fig. 1). A region with a high probability of connection to LOFC (yellow throughout all figures) was apparent in medial MD but with a limited dorso-ventral extent. The region with a high probability of connection to DPFC/VLPFC (blue) occupied a larger area in lateral MD, extending to the ventral limit of MD as predicted. On close inspection, the MD region with a high probability of connection with DLPFC (green) overlapped with the area with a high probability of connection with DPFC/VLPFC and particularly the area with a high probability of connection with LOFC. DLPFC projections were confined to the dorsal portion of MD. Finally, a region with a high probability of connection with ACC (pink) in macaque extended through a broad region in dorsolateral MD.

### Comparison between human and macaque tractography results

**Fig. 5:**
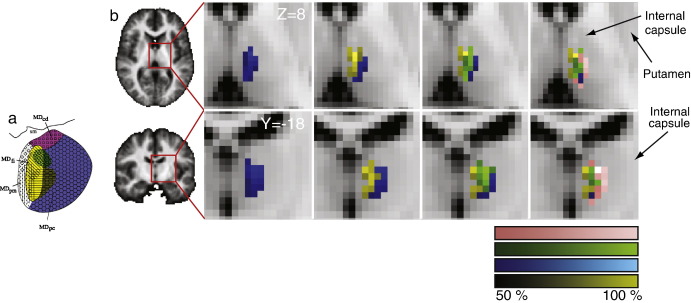
Tractography results in human brain. (a) Results from previous tracer study by Ray and Price (1993) repeated from Fig. 1 for reference. (b) Group results of connectivity-based parcellation in human MD, the upper row depicting transaxial (*Z* = 6), the lower depicting coronal (*Y* = − 18) sections. Voxels within MD are color-coded according to their probability of connection with different cortical areas (shown in Fig. 3). Results are shown in MNI space.

The topography of MD frontal connections in human subjects resembled that seen in the macaque. Fig. 5 depicts mean connectivity likelihood to each of the target regions discussed above, with scaling normalized to the maximum connectivity likelihood observed for each individual target mask.

Table 1 summarizes variability of connectivity to the different cortical targets. Repeated measures ANOVAs were performed on the *X*, *Y*, and *Z* coordinates of locations with highest probability of connectivity to different cortical targets in individual subjects in order to test for evidence of significant topography. We found significant differences in the location of MD regions with a high probability of connection with DPFC/VLPFC, with ACC, with DLPFC, and with LOFC for the medial–lateral axis (*F* = 5.27, *df* = (3,21), *p* = 0.007) and the caudal–rostral axis (*F* = 4.13, *df* = (3,21), *p* = 0.019). Follow up *t*-tests were performed to explore the topographic mapping of connections with different cortical areas and are discussed in detail below.

#### MD connections with LOFC

Regions with strong evidence of connectivity with LOFC were found at the medial aspect of MD in both humans (Fig. 5) and macaque (Fig. 4), in a region likely to correspond to MDfi. In humans, the region with a high probability of connection with LOFC was largely medial to the regions with high probability of connection to DPFC/VLPFC (*t* = 5.23, *df* = 7, *p* = 0.001), and ACC (*t* = 3.42, *df* = 7, *p* = 0.011), was largely rostral to the region with high probability of connection with ACC (*t* = 3.38, *df* = 7, *p* = 0.012) and tended to be rostral to the region with high probability of connection with DLPFC (*t* = 1.93, *df* = 7, *p* < 0.095).

#### MD connections with ACC

In macaque, connectivity to ACC was found in a broad region in caudal and dorsal MD (Fig. 4). In humans, we found a region with a caudal, dorsal and lateral position within MD with strong evidence of connection with ACC (Fig. 5). On visual inspection the center of connectivity was located caudal to the other target regions in all subjects. Follow up *t*-tests confirmed that connectivity for ACC was statistically significantly more caudal and lateral than that for LOFC, as reported above. ACC connectivity was centered on a region that was caudal to the DPFC/VLPFC region (*t* = 4.03, *df* = 7, *p* = 0.005) and slightly more caudal than the area identified with DLPFC (*t* = 2, *df* = 7, *p* = 0.086).

#### MD connections with areas DPFC/VLPFC

As predicted by the macaque DWI study (Fig. 4) and previous tract tracing studies (Fig. 1), there was evidence for a region of human MD with a high probability of connection with DPFC/VLPFC, extending to the ventral limit of MD (Fig. 5). Its location in lateral MD meant that it was likely to correspond to MDpc (Johansen-Berg et al., 2005; Schaltenbrand et al., 1977). As already described above *t*-tests on individual subject coordinates for peak connectivity voxels established that the region with a high probability of connection with DPFC/VLPFC was located more laterally than the region with a high probability of connection to LOFC and more caudally than the ACC region.

#### MD connections with DLPFC

Macaque tract tracing studies have suggested that there is no MD territory exclusive to DLPFC projections. Rather, the thalamic representation shares its location with both areas projecting to DPFC/VLPFC and LOFC (Fig. 1).

The macaque DWI–DT data presented above (Fig. 4) suggested that connections to DLPFC are, within MD, confined to its dorsal part. As already mentioned, in human subjects, the probable DLPFC projection zone is located rostral to the regions with high probability of connection to ACC and LOFC but was not situated in a significantly different MD region to the one identified with DPFC/VLPFC (Fig. 5). The more lateral part of the probable DLPFC projection zone overlaps with the probable DPFC/VLPFC connecting region.

## Discussion

In some primates such as the macaque there is evidence for the existence of at least two networks of interconnected frontal cortical areas centered on the lateral orbitofrontal cortex (LOFC) and medial frontal and anterior cingulate cortex (ACC) (Ongur and Price, 2000). This difference in anatomical connectivity may underlie differences in function between ACC and LOFC (Rushworth et al., 2007a). Some of the first evidence for the existence of the two circuits in the macaque came from a comparison of the relative positions of zones within the MD thalamus projecting to different frontal regions (Ray and Price, 1993). The same study and others (Barbas et al., 1991; Giguere and Goldman-Rakic, 1988; Goldman-Rakic and Porrino, 1985) suggested the existence of a third region of prefrontal cortex, lateral prefrontal cortex (including ventrolateral prefrontal cortex, VLPFC, and dorsal prefrontal cortex, DPFC), with a distinct MD connection pattern; lateral, parvicellular parts of MD (MDpc) project most strongly to lateral prefrontal cortex. Ray and Price (1993) also reported the existence of a fourth region within the MD thalamus that projected mainly to just a limited dorsolateral part of the lateral prefrontal cortex (DLPFC), centered on area 46. The aim of the present study was to test for evidence of topographic differences in human MD–prefrontal connections. Any evidence for such a separation would be consistent with the existence of differences in anatomical connectivity between prefrontal systems in the human brain resembling those found in other primates.

Previous studies have suggested that DWI–DT can be used to distinguish the connections of different thalamic nuclei (Behrens et al., 2003a; Johansen-Berg et al., 2005) but whether the technique is sensitive to differences in connectivity across sub-regions of a single thalamic nucleus has not been investigated. The second and related aim of the present study was, therefore, to test whether DWI–DT could be used to provide evidence for regional variations in connectivity within a single thalamic nucleus — MD.

Using DWI–DT we found clear evidence for topographic difference in connectivity with four frontal regions within the MD nucleus in a macaque. Regional differences in connectivity resembled those previously reported in tracer injection and tract tracing studies. LOFC had a greater probability of interconnection with a relatively medially situated and dorso-ventrally oriented strip of MD thalamus — a pattern that resembled that seen in the macaque (Barbas et al., 1991; Giguere and Goldman-Rakic, 1988; Goldman-Rakic and Porrino, 1985). VLPFC/DPFC had a high probability of interconnection with more lateral MD (Figs. 4 and 5) while a DLPFC region in the principal sulcus that was probably mainly constituted of area 46 had a high probability of connection with a dorsal MD region situated between the first two projection zones. The overall pattern resembled that previously reported in tract tracing investigations in the macaque (Ray and Price, 1993). A fourth region, ACC, was most strongly interconnected with a relatively caudal and lateral part of MD (Figs. 4 and 5). A similar pattern of connectivity between MD and ACC has been reported (Giguere and Goldman-Rakic, 1988).

In humans, DWI–DT produced evidence of MD connections with LOFC that resembled those established in macaque in tract tracing studies (Barbas et al., 1991; Giguere and Goldman-Rakic, 1988; Goldman-Rakic and Porrino, 1985) and using DWI–DT in a macaque brain in the first part of the current study. As in the macaque there was evidence of connections between LOFC and the medial aspect of MD in the human subjects. A previous DWI–DT study also highlighted other similarities between the estimated connection pattern for human LOFC and the pattern established in the macaque; Croxson et al. (2005) reported that the uncinate fascicle had a higher probability of connection with the LOFC than with medial frontal cortex/ACC, or lateral prefrontal cortex in both macaque and human subjects. In the macaque, temporal and perirhinal cortical areas are strongly interconnected with LOFC (Carmichael and Price, 1995a; Kondo et al., 2003, 2005; Lavenex et al., 2002) via the uncinate fascicle (Ungerleider et al., 1989). These connections may underlie a role for the lateral OFC in assigning reinforcement significance to objects represented by networks of neurons in temporal and perirhinal regions (Murray et al., 2007; Rushworth et al., 2007a). As in the macaque, evidence was found for ACC and LOFC connections with distinct MD zones in the human brain. The separation is consistent with the existence in the human brain of distinct anatomical circuits centered on the ACC and LOFC resembling those seen in other mammal species including rats and macaques (Ongur and Price, 2000; Ray and Price, 1993). In rats and macaques the ACC and LOFC circuits are implicated in the control of distinct aspects of reward-guided behavior (Behrens et al., 2007b; Rushworth et al., 2007a). While the ACC is implicated in reward-guided selection of action, the detection of reward prediction errors, and the generation of novel action strategies, the OFC is concerned with the representation of reward outcomes and with the process of associating stimulus and object representations with reward. The ACC connection region in humans was found in a relatively caudo-dorsolateral part of MD as was also the case in the macaque DWI–DT data and as has been reported previously in the macaque (Giguere and Goldman-Rakic, 1988; McFarland and Haber, 2002). Previous DWI–DT studies have highlighted other similarities between the likely connections of human ACC/medial frontal cortex and the established connections of macaque ACC/medial frontal cortex. For example, DWI–DT studies have suggested that some parts of human ACC/medial frontal cortex are connected with motor and premotor regions (Beckmann et al., 2009) as is the case in the macaque (Barbas and Pandya, 1987; Hatanaka et al., 2003; Luppino et al., 2003; Rizzolatti et al., 1998; Takada et al., 2004). Such connections could explain the greater importance of ACC/medial frontal cortex, as opposed to LOFC, in learning the reinforcement significance of actions in several species (Ostlund and Balleine, 2007; Rudebeck et al., 2008; Rushworth et al., 2007a). Other connections of ACC/medial frontal cortex, with areas such as the hypothalamus and amygdala, may underlie its importance in social cognition in rats, monkeys, and humans (Beckmann et al., 2009; Behrens et al., 2009, 2008; Rudebeck et al., 2006, 2007).

Evidence for connections with DPFC and VLPFC, prefrontal regions involved with memory retrieval, conditional learning, language, and action selection (Murray et al., 2000; Passingham et al., 2000; Petrides, 2005), was found in lateral MD, again matching predictions from macaque. The MD region with a high probability of connection with the DLPFC in the middle frontal gyrus, in between the VLPFC and DPFC, was situated between the MD regions interconnected with LOFC on the one hand and VLPFC and DPFC on the other hand. This pattern is reminiscent of the DLPFC connection pattern in the macaque seen in the present study and in previous reports (Ray and Price, 1993). Again there is increasing evidence for important similarities between the connection patterns of lateral frontal regions and temporal and parietal cortical areas via the extreme capsule and branches of the superior longitudinal fascicle in macaques and humans (Croxson et al., 2005; Frey et al., 2008; Rilling et al., 2008).

In our approach, we use macroscopic anatomy to define cortical targets for DWI–DT, while in macaque, projections from MD have been established with respect to cytoarchitectonically defined regions (Ray and Price, 1993). In our study on living human brains, cytoarchitectonic data on an individual level are not available. There is an approximate relationship between macroscopic features such as sulci and gyri and cytoarchitectonic anatomy but it is not always exact (Amunts et al., 1999; Fischl et al., 2008). Current developments in MRI may eventually overcome the problem of defining individual cytoarchitectonic borders in the human brain (Walters et al., 2007).

The choice of registration method to account for individual differences in brain size or anatomy might influence the accuracy of the registration of brains to one another (Klein et al., 2009), and hence has some impact on the degree to which parcellation appears similar across subjects. While a non-linear approach can be expected to offer a benefit in registration accuracy, our results demonstrate that linear registration is capable of preserving individual connectivity characteristics of MD sub-portions.

In comparison with tracer injection studies that can be carried out only in animals, DWI–DT suffers from several limitations (Johansen-Berg and Behrens, 2006). DWI–DT has a lower spatial resolution and it cannot identify the polarity of connections. In the case of MD–prefrontal connections it is known from tracer injection studies that regional variation in connectivity patterns is most apparent when the thalamocortical, rather than corticothalamic connections, are considered (McFarland and Haber, 2002). Haber and McFarland reported that while there are corticothalamic projections that reciprocate thalamocortical projections there are, in addition, corticothalamic projections to more extensive regions of thalamus. Because the DWI–DT technique used here is sensitive to relative differences in connection probability, however, it is still able to identify regional variations in MD–prefrontal connectivity.

Importantly, DWI–DT can only be used to establish the *probability* of interconnection between areas rather than providing definitive evidence for the presence of fiber terminals and synapses. A major potential source of bias in tractography is any of a number of strong fiber systems that connections between MD and the frontal lobe would need to traverse before reaching their target. Callosal fibers, the cingulum bundle and parts of the superior longitudinal fascicle could disrupt DWI–DT investigation of connections between MD and the frontal lobe, particularly medial areas such as the ACC (Schmahmann et al., 2007). Nevertheless, the similarity between the DWI–DT and tract tracing results suggests that the current algorithm, which is able to identify multiple orientations of fibers in any voxel (Behrens et al., 2007a), was able to identify and follow the major pathways between MD and frontal cortex. It is, however, important to remember that for the human data acquired here (with 60 diffusion encoding directions), it is not possible to resolve more than two different fiber populations in any voxel (Behrens et al., 2007a). Despite these disadvantages DWI–DT is important because it makes it possible to investigate whether neuroanatomical connection patterns found in experimental animals, such as monkeys, are also likely to be present in humans.

In summary, DWI–DT confirms that the principal features of connectivity between MD and anterior cingulate and prefrontal cortex in humans are comparable to those found in macaque using DT–DWI or tract tracing techniques. There is a clear separation between MD regions with a higher probability of interconnection with ACC or LOFC consistent with the possibility that there are distinct anatomical circuits centered on ACC and LOFC concerned with distinct aspects of reward-guided behavior. Importantly this is the first study to provide insight into the topography of projections of an individual thalamic nucleus, and demonstrates the value of DWI–DT as a technique to study human anatomy. While tracer techniques remain the gold standard of establishing connectivity between brain structures, DWI–DT's non-invasive nature enables its use for whole brain studies in human subjects. DWI–DT can be used to examine the degree to which anatomical interconnectivity patterns established in animal models are likely to pertain to the human brain (Behrens et al., 2003a; Croxson et al., 2005; Ramnani et al., 2006; Rushworth et al., 2006; Tomassini et al., 2007).

## Figures and Tables

**Figure:**
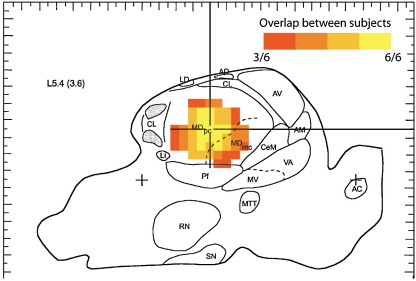
Supplementary figure

**Table 1:** 

Target region	*X* coordinate		*Y* coordinate		*Z* coordinate	
Mean	SD	Mean	SD	Mean	SD
LOFC	− 5.8	1.67	− 14.7	2.82	7.5	4.11
ACC	− 8.3	1.67	− 20.0	2.83	8.3	3.11
DPFC/VLPFC	− 8.5	0.93	− 15.5	3.34	7.5	2.33
DLPFC	− 7.3	1.49	− 16.0	4.54	8.3	4.33

Mean coordinate and standard deviation (SD) of the peak voxel of connectivity to individual target regions. Coordinates reported in mm relative to the ICBM-152 brain template's anterior commissure.
